# A polygeneric immunogen composed of 22 venoms from sub-Saharan African snakes to expand the neutralization scope of the EchiTAb-plus-ICP antivenom

**DOI:** 10.1016/j.toxcx.2024.100213

**Published:** 2024-11-16

**Authors:** Andrés Sánchez, Gina Durán, Maykel Cerdas, Jairo Gutiérrez, Álvaro Segura, María Herrera, Mariángela Vargas, Adriana Sánchez, Paola Sánchez, Gabriela Solano, Mauren Villalta, Edwin Moscoso, Deibid Umaña, Mauricio Arguedas, Aarón Gómez, José María Gutiérrez, Guillermo León

**Affiliations:** Instituto Clodomiro Picado, Facultad de Microbiología, Universidad de Costa Rica, San José, Costa Rica

**Keywords:** Cross-neutralization, EchiTAb-plus-ICP, Horse immunization, Snake antivenom, Snake venom

## Abstract

Recent research suggests that a polygeneric immunogen made from the venoms of the most medically important viperid and elapid snakes in sub-Saharan Africa could elicit a broader antibody response in horses compared to the current EchiTAb-plus-ICP antivenom, especially against neurotoxic elapid venoms. To test this, 25 horses that have been regularly immunized to produce this antivenom were reimmunized with an immunogen containing 22 venoms from various snake species from the genera *Bitis*, *Echis*, *Dendroaspis*, and both spitting and non-spitting *Naja*. The plasma collected from these horses was processed using the caprylic acid method to produce an industrial-scale freeze-dried antivenom. The anti-lethal neutralization scope of this new formulation was then compared to that of EchiTAb-plus-ICP which is designed to target the venoms of *Bitis arietans*, *Echis ocellatus*, *Naja nigricollis*, and *Dendroaspis polylepis*. The results indicated that adding more venoms to the immunogen did not significantly enhance the neutralization of the lethal effect of viperid venoms (except for *Bitis nasicornis*) or of venoms of spitting cobras (except for *Naja katiensis*). However, incorporating additional venoms from non-spitting neurotoxic *Naja* spp. and *Dendroaspis* spp. improved the neutralization scope of EchiTAb-plus-ICP against these neurotoxic venoms. The antivenom generated showed a wider anti-lethal neutralizing scope, as compared to the standard EchiTAb-plus-ICP antivenom and constitutes a good candidate to be tested in clinical trials in sub-Saharan Africa.

## Highlights

• An immunogen composed of 22 snake venoms was used to immunize 25 horses.
• The venoms were derived from snakes belonging to the *Bitis*, *Echis*, *Dendroaspis* and *Naja* genera.
• An antivenom was made from the plasma of these horses by the caprylic acid method.
• The antivenom exhibited a broader neutralizing scope compared to EchiTAb-plus-ICP.

## Graphical abstract

**Figure:**
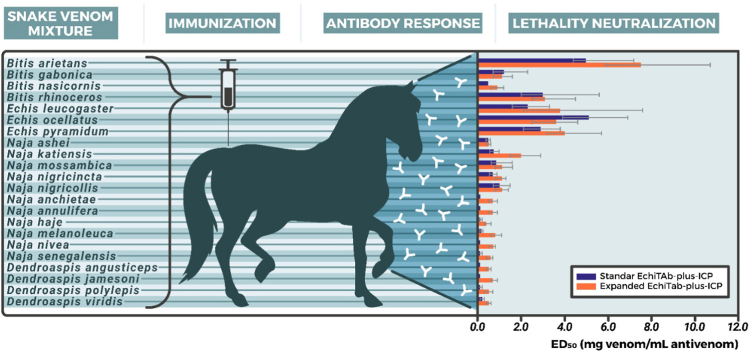

## 1 Introduction

Antivenoms are the only medicines whose suitability for the treatment of snakebite envenomation has been demonstrated in properly designed clinical trials (WHO, 2017). The active substances of antivenoms are immunoglobulins, or their F(ab’)_2_ or Fab fragments, purified from the plasma of animals immunized with snake venoms (WHO, 2017). These antibodies can recognize and neutralize not only the venoms used as immunogens (i.e., homologous venoms) but also other antigenically similar venoms (i.e., heterologous venoms) of phylogenetically related snakes (León et al., 2011).

The medical significance of snakes in a specific region is a primary consideration for selecting venoms used as immunogens for antivenom production. In sub-Saharan Africa, the *Bitis* spp. (Puff-adders), *Echis* spp. (Carpets), *Naja* spp. (spitting and non-spitting cobras), and *Dendroaspis* spp. (Mambas) are of major medical importance (WHO, 2017). Consequently, the venoms from snakes belonging to these genera are used as immunogens in the production of commercially available antivenoms in this region (Brown, 2012; Sánchez et al., 2015; Ainsworth et al., 2020; Offor et al., 2022).

Species of each snake genus have venoms with a specific composition (Offor et al., 2022) that allow them to produce envenomations with characteristic clinical manifestations corresponding to cytotoxic, hemotoxic, or neurotoxic syndromes (WHO, 2010). Identifying the type of envenomation in sub-Saharan Africa can be done by following a syndrome-based approach. However, the similarity in clinical presentations of envenomations caused by sympatric snake species, combined with the limited training of some healthcare personnel and the lack of specific diagnostic tests, can lead to the misidentification of the responsible snake and the selection of inappropriate antivenom, ultimately resulting in treatment failure. This issue is not limited to sub-Saharan Africa but is a global concern (Bolon et al., 2020). Consequently, some physicians and health authorities prefer polyspecific pan-African-like antivenoms capable of neutralizing the venoms of various genera causing diverse syndromes.

The production of this type of antivenom presents the challenge of identifying the venoms that give the widest coverage of species when used as immunogens. As each producer has devised its own solution to this problem (Brown, 2012; Ainsworth et al., 2020; Offor et al., 2022), a variety of neutralizing profiles is observed among different antivenom formulations (Dalhat et al., 2023; Ainsworth et al., 2020; Offor et al., 2022). It is necessary to identify the optimum venom mixture to generate pan-African antivenoms of wide neutralization spectrum on the basis of empirical evidence.

In recent studies, the antigenic similarity between venoms of sub-Saharan snakes of the same genus was inferred from the cross-reactivity of monospecific antisera produced in rabbits (Gómez et al., 2022, 2023, 2024). It was found that the venoms with the major antigenic representation in their corresponding genera were those of *Bitis gabonica*, *Echis leucogaster*, *Naja nigricollis*, *Naja senegalensis*, and *Dendroaspis polylepis*. Presumably, monospecific antivenoms produced towards these venoms should have the widest intrageneric neutralization scope.

This presumption was tested by comparing the intrageneric neutralization scope of sera from horses immunized with monospecific, bispecific/monogeneric, or polyspecific/monogeneric venom mixtures. Results indicated that the polyspecific immunogens tend to be a better option to induce antibody responses with a broader neutralization scope (Sánchez et al., 2024a,b). Based on these results, it was hypothesized that a combination of polyspecific immunogens from *Bitis* spp., *Echis* spp., spitting and non-spitting *Naja* spp., and *Dendroaspis* spp. may trigger an antibody response in horses able to neutralize the venoms of a large number of medically-relevant species in sub-Saharan Africa and would have a wider neutralizing scope than that of the standard EchiTAb-plus-ICP, which is produced with an immunogen composed of only four venoms (i.e., *Bitis arietans*, *Echis ocellatus*, *Naja nigricollis*, and *Dendroaspis polylepis*).

To test this hypothesis, a group of horses was immunized with a mixture of venoms from 22 species of sub-Saharan African snakes belonging to the genera *Bitis*, *Echis*, *Dendroaspis*, and *Naja*. The hyperimmune plasma was processed using the caprylic acid method to produce an industrial-scale batch of a freeze-dried formulation of the expanded EchiTAb-plus-ICP antivenom. The neutralization scope of this new formulation was then analyzed and compared with that of the standard EchiTAb-plus-ICP antivenom.

## 2 Materials and methods

### 2.1 Ethics

The study procedures were approved by the Institutional Committee for the Care and Use of Laboratory Animals (CICUA) at the University of Costa Rica (Proceedings 82-08 and 39-20) and were in accordance with the International Guiding Principles for Biomedical Research Involving Animals (CIOMS, 2012). All animal procedures were conducted following animal welfare guidelines to ensure their well-being and minimize any potential distress or harm. Twenty-five horses were kept in paddocks at a farm located at 1495 AMSL, with access to pasture and water *ad libitum*. The horses were provided with pelleted feed containing proteins, vitamins, and minerals. Immunization and blood collection procedures were overseen by a veterinarian in a specialized facility on the same farm. Mice were obtained from the Bioterium of Instituto Clodomiro Picado and handled in Tecniplast Eurostandard Type II 1264C cages, five mice per cage, at 18–24 °C, 60–65% relative humidity, and 12:12 light-dark cycle, with food and water *ad libitum*.

### 2.2 Venoms

Freeze-dried venoms from adult specimens of several snake species were purchased from Latoxan (Portes-lès-Valence, France): *B. arietans*, *B. gabonica*, *Bitis nasicornis*, *Bitis rhinoceros*, *E. leucogaster*, *E. ocellatus*, *Echis pyramidum*, *Naja ashei*, *Naja katiensis*, *Naja mossambica*, *Naja nigricincta*, *N. nigricollis*, *Naja anchietae*, *Naja annulifera*, *Naja haje*, *Naja melanoleuca*, *Naja nivea*, *N. senegalensis*, *Dendroaspis angusticeps*, *Dendroaspis jamesoni*, *D. polylepis*, and *Dendroaspis viridis*. Solutions of the venoms were prepared immediately before use. The serial numbers of venom batches and their geographical origins are detailed in Table 1.

**Table 1:** Median lethal dose (LD_50_) of venoms, determined by the intravenous route and expressed as μg venom per mouse^a^.

Venom	Batch^b^	Geographical origin	LD_50_ (95% CI)
*Bitis arietans*	#322.061	unspecified origin,	11.4 (8.5–15.7)
*Bitis gabonica*	#725.031	unspecified origin,	20.6 (18.1–23.5)
*Bitis nasicornis*	#500.102	unspecified origin,	19.8 (17.2–22.9)
*Bitis rhinoceros*	#701.070	Ghana	17.9 (15.1–20.4)
*Echis leucogaster*	#623.070	Mali	29.9 (25.7–34.7)
*Echis ocellatus*	#216.031	unspecified origin,	18.3 (17.0–19.7)
*Echis pyramidum*	#523.070	Egypt	12.9 (9.4–17.4)
*Dendroaspis angusticeps*	#305.000	Tanzania/Mozambique	29.5 (25.9–33.5)
*Dendroaspis jamesoni*	#923.011	Cameroon	21.3 (19.6–24.9)
*Dendroaspis polylepis*	#416.031	unknown origin	6.5 (6.0–7.3)
*Dendroaspis viridis*	#516.001	Ghana/Togo	10.9 (10.1–11.9)
*Naja anchietae*	#527.002	Namibia	51.6 (34.9–78.1)
*Naja annulifera*	#622.040	Mozambique	48.1 (42.6–54.2)
*Naja ashei*	#410.191	Kenya	18.4 (12.9–26.1)
*Naja haje*	#222.061	unknown origin,	11.7 (9.3–17.9)
*Naja katiensis*	#705.010	Burkina Faso	18.4 (13.7–24.7)
*Naja melanoleuca*	#516.031	unknown origin	6.9 (5.3–9.3)
*Naja mossambica*	#627.002	Tanzania	20.8 (18.2–24.0)
*Naja nigricincta*	#507.081	South Africa	16.2 (14.0–18.5)
*Naja nigricollis*	#616.031	unknown origin,	19.8 (17.7–22.4)
*Naja nivea*	#524.010	South Africa	33.4 (28.7–38.6)
*Naja senegalensis*	#805.010	Mali	10.4 (7.3–13.4)

a Values in parentheses represent the 95% confidence intervals (CI).

b Batch numbers of Latoxan.

### 2.3 Determination of median lethal dose (LD_50_)

Groups of five mice (16–18 g; CD-1 strain, both sexes) were given a subcutaneous injection of the analgesic Tramadol (50 mg/kg) to alleviate pain and discomfort during the experiment (Chacón et al., 2015). After a 15-min interval, mice received an intravenous (IV) injection of 0.2 mL of a 0.12 M NaCl, 0.04 M phosphate solution (PBS) at pH 7.2, which contained varying amounts of venom. The number of deaths within the next 24 h was recorded to calculate the median lethal dose (LD_50_), which is the quantity of venom that causes the death of 50% of the treated mice, using Probits analysis (Finney, 1971). The surviving mice were euthanized by CO_2_ inhalation. The findings were presented as LD_50_ along with the associated 95% confidence interval (95% CI).

### 2.4 Immunization and industrial bleeding

Over a period of at least three years, 25 horses (of no defined breed, weighing 350–450 kg) were routinely immunized with a venom mixture consisting of venoms from *B. arietans*, *E. ocellatus*, *N. nigricollis*, and *D. polylepis*. They were then subjected to industrial bleeding to produce plasma for the manufacturing of the standard antivenom EchiTAb-plus-ICP (Gutiérrez et al., 2005; Huertas et al., 2023). Subsequently, the venom mixture was altered to include equal parts per genus of a mixture of 22 venoms from the species belonging to *Bitis*, *Echis*, spitting and non-spitting *Naja*, and *Dendroaspis* genera to generate the expanded EchiTAb-plus-ICP antivenom. The *Bitis* spp. component comprised equal amounts of venoms from *B. arietans*, *B. gabonica*, *B. nasicornis*, and *B. rhinoceros*. The *Echis* spp. component included equal amounts of venoms from *E. leucogaster*, *E. ocellatus*, and *E. pyramidum*. The spitting *Naja* spp. component consisted of equal amounts of venoms from *N. ashei*, *N. katiensis*, *N. mossambica*, *N. nigricincta*, and *N. nigricollis*. The non-spitting *Naja* spp. component was composed of equal amounts of venoms from *N. anchietae*, *N. annulifera*, *N. haje*, *N. melanoleuca*, *N. nivea*, and *N. senegalensis*. The *Dendroaspis* spp. component was made up of equal amounts of venoms from *D. angusticeps*, *D. jamesoni*, *D. polylepis*, and *D. viridis*. The venom mixture was dissolved in physiological saline solution, sterilized through 0.22 μm filtration, and dispersed in a similar volume of the mineral oil adjuvant Montanide ISA 50 V 2 to create a simple w/o emulsion (Arguedas et al., 2022). For two and a half months, every two weeks, the horses were injected subcutaneously at a single site on the horse's back with 1.0 mL of the immunogenic emulsion containing 1.0 mg of the new venom mixture (i.e., 0.2 mg of each genus mixture).

Ten days after the final venom booster, the horses underwent physical and hematological assessments to ensure that only those in optimal condition were selected for industrial bleeding. On the 12th day, 6–8 L of blood were collected from the jugular vein of each horse using a system of PVC blood bags. An anticoagulant solution of citrate dextrose (ACD; 0.093 mol/L citric acid, 0.197 mol/L sodium citrate, and 0.6 mol/L dextrose) was used. The blood bags were stored at 2–8 °C. Two weeks later, the red blood cells (RBCs) were separated from plasma, suspended in 3 L of saline solution, and warmed to 37 °C. The horses then underwent another industrial bleeding session. Immediately after collecting 6–8 L of blood, horses were self-transfused with their resuspended RBCs from the previous bleeding session. The new blood bags were stored at 2–8 °C for two weeks. Two days later, the horses were reimmunized to begin the bleeding cycle again (Arias-Esquivel et al., 2024). The entire process was carried out under veterinary supervision.

### 2.5 Immunoglobulin purification and antivenom formulation

Four hundred kilograms of undiluted plasma were precipitated, under vigorous stirring, by the slow addition of caprylic acid until a final concentration of 4.0% (v/v) was achieved. The precipitated material was removed by dynamic body-feed filtration (Filtrodisc Bio SD 16” double module, CH09P; Filtrox AG, St. Gallen, Switzerland) assisted with diatomite (Celpure®, C1000; Filtrox AG, St. Gallen, Switzerland; Sánchez et al., 2024a,b). The filtrate underwent five diafiltration cycles using 18.6 m^2^ of a 30 kDa cutoff ultrafiltration membrane (CDUF050LT, Helicon® SS50 Spiral Cartridge, Millipore, Darmstadt, Germany). The diafiltrated product was formulated at pH 7.2, 0.30–0.35 g/dL NaCl, 0.15–0.20 g/dL phenol, and 4.0–5.0 g/dL sucrose and total protein of 6.5 g/dL. The formulated product was sterilized by filtration through a 0.22 μm filter (5102507H2; Sartorius; Göttingen, Germany), and filled into 10 mL borosilicate type 1 vials. Finally, the antivenom was stabilized by freeze-drying (frozen at −40 °C, annealed at −10 °C for 4 h, sublimed at a shelf temperature of −10 °C and a chamber pressure of 200 mTorr for 64 h, and desorbed at 30 °C for 4 h; Herrera et al., 2017). The standard antivenom EchiTAb-plus-ICP (batch 6771021PALQ, expiry date October 2024) was used as a reference for comparisons.

### 2.6 Determination of median effective dose (ED_50_)

Mixtures of a fixed amount of venom and various dilutions of antivenom were prepared and then incubated at 37 °C for 30 min. Subsequently, 0.2 mL of each mixture, containing a venom challenge dose equivalent to 3 LD_50_ for elapids or 5 LD_50_ for viperids, was injected into groups of five CD-1 mice (16–18 g) by the IV route. Controls included mice receiving venom incubated with PBS instead of antivenom. Before injection of venom or venom-antivenom mixtures, mice were pre-treated with the analgesic tramadol, administered subcutaneously at a dose of 50 mg/kg (Chacón et al., 2015). The number of deaths in each group was recorded 24 h after injection, and the surviving mice were euthanized by CO_2_ inhalation. ED_50_ was estimated by Probits (Finney, 1971) and expressed as the ratio mg venom/mL antivenom at which half of the injected mice survived. Results were expressed as ED_50_ and the corresponding 95% confidence interval (95% CI). An observation time of 24 h was used in these experiments because it is the time interval indicated for neutralization tests using the IV injection in the WHO antivenom guidelines (WHO, 2017), and the one generally required by regulatory authorities for product registration.

### 2.7 Statistical analyses

The values of ED_50_s were considered significantly different when the 95% CI did not overlap.

### 2.8 Physicochemical and microbiological quality control

Residual moisture was assessed through Karl Fisher titration (Herrera et al., 2014). The reconstitution time was visually evaluated as the duration required for the lyophilized antivenom to fully dissolve with gentle hand agitation (Herrera et al., 2014). Turbidity was measured using turbidometry (Model, 2020 Turbidimeter, La Motte, USA) and expressed in nephelometric turbidity units (NTU). Residual caprylic acid was analyzed following the HPLC method outlined by Herrera et al. (2009). pH was determined using potentiometry (USP-NF, 2024a). The NaCl content was determined through a volumetric method (USP-NF, 2024b). Phenol concentration was assessed using the 4-aminoantipyrine spectrophotometric technique (Lacoste et al., 1959). Sucrose levels were analyzed using HPLC with refractometry detection (Agilent 1220 Infinity II HPLC with 1260 Infinity II RID module, Zorbax NH2 column). The osmotic concentration of solutes in the antivenoms was measured using osmometry (Advanced Instruments, Model 3320). Total protein content was evaluated by the Biuret method (Gornall et al., 1949). Sterility was evaluated using the membrane filtration method (USP-NF, 2024c), where membranes were cultured in thioglycolate and soy trypticase media. Endotoxins were assessed using the Limulus amebocyte lysate (LAL) assay (USP-NF, 2024d; Solano et al., 2015).

## 3 Results and discussion

Currently, several commercial antivenoms are distributed in various regions of sub-Saharan Africa. The manufacturers of these products use different mixtures of venoms to elicit an antibody response in the horses used as a source of immunoglobulins. They also formulate their products with varying total protein contents. Consequently, the scope of neutralization provided by these antivenoms varies from one product to another (Dalhat et al., 2023; Ainsworth et al., 2020; Offor et al., 2022).

**Fig. 1:**
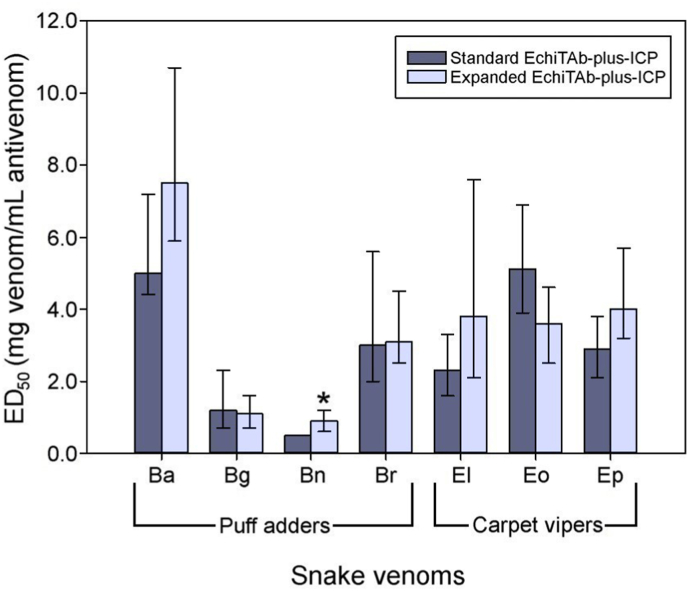
Neutralizing ability of the lethal effect of viperid venoms by the standard EchiTAb-plus-ICP antivenom and the expanded EchiTAb-plus ICP antivenom prepared by immunization with a mixture of venoms of 22 species. The venoms tested were *B. arietans* (Ba), *B. gabonica* (Bg), *B. nasicornis* (Bn), *B. rhinoceros* (Br), *E. leucogaster* (El), *E. ocellatus* (Eo), and *E. pyramidum* (EP). ∗ Significant differences exist when the ED_50_ values have non-overlapping 95% CI. Numerical values are presented in Supplementary Table S1.

**Fig. 2:**
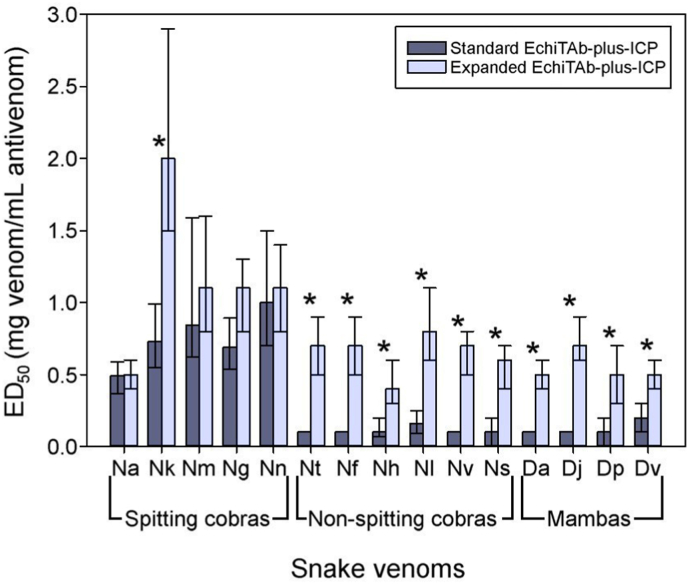
Neutralizing ability of the lethal effect of elapid venoms by the standard EchiTAb-plus-ICP antivenom and the expanded EchiTAb-plus ICP antivenom prepared by immunization with a mixture of venoms of 22 species. The tested venoms were *N. ashei* (Na), *N. katiensis* (Nk), *N. mossambica* (Nm), *N. nigricincta* (Ng), *N. nigricollis* (Nn), *N. anchietae* (Nt), *N. annulifera* (Nf), *N. haje* (Nh), *N. melanoleuca* (Nl), *N. nivea* (Nv), *N. senegalensis* (Ns), *D. angusticeps* (Da), *D. jamesoni* (Dj), *D. polylepis* (Dp), and *D. viridis* (Dv). ∗ Significant differences exist when the ED_50_ values have non-overlapping 95% CI. Numerical values are presented in Supplementary Table S1.

In the case of the standard EchiTAb-plus-ICP, the plasma used as raw material is collected from horses originally immunized against the venoms of *B. arietans*, *E. ocellatus*, and *N. nigricollis* (Gutiérrez et al., 2005). More recently, the venom of *D. polylepis* was included in the immunization mixture. We tested the ability of this antivenom to neutralize venoms from a variety of medically relevant viperid and elapid venoms of sub-Saharan Africa. Regarding the neutralization of viperid venoms, the standard EchiTAb-plus-ICP was effective against venoms of *Bitis* sp and *Echis* sp, albeit with variable ED_50_s, with the exception of *B. nasicornis* (Fig. 1; Supplementary Table S1). In the case of elapid venoms, the neutralizing scope of the standard EchiTAb-plus-ICP varied depending on whether venoms correspond to spitting cobras or non-spitting cobras and mambas. As shown in Fig. 2 and Supplementary Table S1, the antivenom was effective in the neutralization of cytotoxic spitting cobra venoms. In contrast, in the case of neurotoxic *Naja* sp and *Dendroaspis* sp venoms, the antivenom either failed to neutralize them or showed a low neutralizing efficacy. These results are similar to those reported in previous studies (Segura et al., 2010; Petras et al., 2011; Sánchez et al., 2017).

On this basis, the main goal of our study was to assess whether the addition of more viperid and elapid venoms to the immunizing mixture used to generate EchiTAb-plus-ICP would improve the neutralizing scope of the antivenom. Results show that the addition of more viperid, i.e. *Bitis* sp and *Echis* sp, venoms to the immunogen mixture did not result in significant improvements in the neutralization of lethality in the venoms of various species of these genera, except for *B. nasicornis* (Fig. 1; Supplementary Table S1).

These results suggest that the toxins responsible for the lethal activity in mice across the *Bitis* spp. and *Echis* spp. genera are adequately represented in the venoms of *B. arietans* and *E. ocellatus*, respectively. These findings disagree with previous results based on a rabbit model where venoms of *B. arietans* and *E. ocellatus* elicited antisera with limited cross-neutralizing activity against congeneric venoms (Gómez et al., 2022). Furthermore, our results challenge the conclusions of a previous study in horses suggesting that the use of polyspecific immunogens elicits higher antibody responses in horses compared to monospecific immunogens in the cases of *Echis* sp and *Bitis* sp venoms (Sánchez et al., 2024a,b).

The differences between the predictions made in earlier studies and our present experimental findings suggest that factors other than the antigenic similarity of venoms influence their capacity to trigger antibody responses with a wide neutralization scope. Previous research has shown that specific venoms in polyspecific immunogens can modify the antibody response triggered by co-immunogen venoms (Arroyo et al., 2015, 2017; Alfaro-Chinchilla et al., 2021). Further investigation is needed to determine if such a phenomenon occurs in the 22 venoms immunogen mixture used to expand the neutralization scope of EchiTAb-plus-ICP. Moreover, an optimal immunization approach may not only involve periodically administering a single venom mixture to horses but also immunizing the same horses with different venom mixtures at varying intervals or immunizing separate groups of horses with different venom mixtures and then combining their plasma or purified immunoglobulins.

The immunogen composed of 22 venoms does not improve the ability of EchiTAb-plus-ICP to neutralize the venoms of spitting cobras, except for *N. katiensis* (Fig. 2; Supplementary Table S1). This result suggests that the toxins responsible for the lethal activity in mice across the spitting *Naja* spp. are adequately represented in the venom of *N. nigricollis*. This is in line with predictions from a rabbit model (Gómez et al., 2023). Additionally, this finding aligns with previous observations indicating that a monospecific immunogen composed of *N. nigricollis* venom generates an antiserum in horses with similar neutralizing efficacy against spitting cobra venoms, as compared with one generated by immunization with several venoms (Sánchez et al., 2024a,b).

The standard EchiTAb-plus-ICP was weak or ineffective in neutralizing the lethal effects of venoms from neurotoxic non-spitting cobras (Fig. 2; Supplementary Table S1). This outcome was expected since the immunogen used to produce this antivenom does not include venoms of these snakes. Although there is antigenic similarity among the venoms of non-spitting cobras (Gómez et al., 2023), a polyspecific immunogen has been identified as the most effective option for eliciting antibody responses that can broadly neutralize these venoms (Sánchez et al., 2024a,b). Consistent with this, using an immunogen that incorporates several venoms from non-spitting cobras generated an antivenom that effectively neutralized the lethality caused by all venoms of neurotoxic cobras tested (Fig. 2; Supplementary Table S1).

The standard EchiTAb-plus-ICP antivenom was ineffective or showed a low value of ED_50_ when tested against venoms of *Dendroaspis* sp. (Fig. 2; Supplementary Table S1). This finding is consistent with the low anti-mamba response observed in rabbits immunized with the *D. polylepis* venom (Gómez et al., 2024). The inclusion of additional mamba venoms in the immunogen expanded the antivenom's anti-*Dendroaspis* neutralization scope (Fig. 2; Supplementary Table S1). This aligns with a previous observation that the antibody response induced by a mamba polyspecific immunogen tends to be higher than that induced by monospecific or bispecific immunogens (Sánchez et al., 2024a,b).

One limitation of this study, in relation to viperid venoms, is that only the neutralization of the lethal effect was evaluated. Since these venoms also induce hemorrhagic activity and, in the case of *Echis* sp venoms, *in vitro* procoagulant effect, it would be necessary to assess whether the antivenom generated with 22 venoms has a higher neutralizing ability against these additional effects as compared to the current EchiTAb-plus-ICP antivenom.

On the other hand, it should be kept in mind that the mouse lethality model does not necessarily predict the efficacy of an antivenom in the clinical setting (Marriott et al., 2024). The efficacy of antivenoms can only be ascertained through well-designed clinical trials, such as those conducted to evaluate the effectiveness and safety of EchiTAb-plus-ICP in Nigeria (Abubakar et al., 2010a, 2010b; Hamman et al., 2024). Additionally, valuable insights can also be gained from prospective or retrospective observational studies, such as those conducted in Nigeria (Dajel et al., 2023) and Ethiopia (Steegemans et al., 2022). Thus, the observed enhancement of the preclinical neutralizing efficacy against neurotoxic elapid venoms of the antivenom generated by immunization with 22 venoms should be evaluated at the clinical level.

The effectiveness and safety of antivenoms are influenced by both the immunological characteristics of the formulation and their physicochemical properties. Table 2 presents a summary of the quality control results for the batches of standard and expanded EchiTAb-plus-ICP used in this study.

**Table 2:** Physicochemical and microbiological quality of antivenoms.

	Standard	Expanded
	EchiTAb-plus-ICP (Batch 6771021PALQ)	EchiTAb-plus-ICP (Batch 7131223PALF)
Residual moisture (%)	Not applicable	2.7 ± 0.7
Reconstitution time (s)^a^	Not applicable	50 ± 10
Turbidity (NTU)	26 ± 1	24.4 ± 0.3
Residual caprylic acid (ppm)	150 ± 9	117 ± 14
pH	6.9 ± 0.1	6.7 ± 0.1
NaCl (g/dL)	0.87 ± 0.01	0.84 ± 0.01
Phenol (g/dL)	0.23 ± 0.01	0.12 ± 0.01
Sucrose (g/dL)	Not applicable	3.8 ± 0.1
Osmolality (mOsm/kg)	332 ± 4	451 ± 2
Total protein (g/dL)	7.2 ± 0.1	6.5 ± 0.1
Sterility (tioglycolate)	Absence of growth	Absence of growth
Sterility (soy trypticase)	Absence of growth	Absence of growth
Endotoxins (EU/mL)	≤3.0	<3.0

a Reconstitution time was reported as the average ± SD of six independent determinations.

## 4 Final remarks

The use of a polygeneric immunogen made from 22 venoms sourced from sub-Saharan Africa has proven effective in broadening the preclinical neutralization capabilities of the EchiTAb-plus-ICP antivenom, especially in the case of neurotoxic elapid venoms. On the basis of these findings, the antivenom generated by using the new venom mixture is a good candidate to be evaluated in clinical trials in sub-Saharan Africa. Formulating immunogens that elicit antibody responses with a wide neutralization scope is a complex process that extends beyond considering just the medical importance of the snakes and the antigenic similarities among their venoms. Further research is necessary to understand how venoms from African snakes influence the antibody response towards co-immunogen venoms in order to further expand and improve the neutralizing scope of pan-African antivenoms.

On the other hand, the antibody response in horses may be influenced by the method used during the initial immunization with snake venoms, a phenomenon known as “original antigenic sin” (Maltseva et al., 2024). Consequently, the antibody response of a group of horses, such as those involved in this study, which received a mixture of four venoms for three years before transitioning to a mixture of 22 venoms, may differ from that of horses immunized from the outset with a mixture of 22 venoms. The significance of this issue in antivenom production warrants further investigation.

The primary advantage of polygeneric antivenoms with a broad neutralization spectrum is that, theoretically, they can be used in cases where the specific snake responsible for the envenomation has not been identified. However, since the neutralizing efficacy of polygeneric antivenoms varies depending on the type of venom, it is essential to establish a single optimum initial dose. This issue can be addressed by comparing the new antivenom with a reference antivenom that has demonstrated efficacy in clinical trials or by conducting clinical trials specifically designed to determine this initial dose. The definition of the optimum initial dose for the expanded EchiTab-plus-ICP remains to be established.

## CRediT authorship contribution statement

**Andrés Sánchez:** Writing – review & editing, Writing – original draft, Investigation, Conceptualization. **Gina Durán:** Writing – review & editing, Investigation. **Maykel Cerdas:** Writing – review & editing, Investigation. **Jairo Gutiérrez:** Writing – review & editing, Investigation. **Álvaro Segura:** Writing – review & editing, Investigation. **María Herrera:** Writing – review & editing, Investigation. **Mariángela Vargas:** Writing – review & editing, Investigation. **Adriana Sánchez:** Writing – review & editing, Investigation. **Paola Sánchez:** Writing – review & editing, Investigation. **Gabriela Solano:** Writing – review & editing, Investigation. **Mauren Villalta:** Writing – review & editing, Investigation. **Edwin Moscoso:** Writing – review & editing, Investigation. **Deibid Umaña:** Writing – review & editing, Investigation. **Mauricio Arguedas:** Writing – review & editing, Investigation. **Aarón Gómez:** Writing – review & editing, Investigation. **José María Gutiérrez:** Writing – review & editing, Writing – original draft, Project administration, Funding acquisition, Conceptualization. **Guillermo León:** Writing – review & editing, Writing – original draft, Project administration, Funding acquisition, Conceptualization.

## Ethical statement

This study was approved by the Institutional Committee for the Care and Use of Laboratory Animals (CICUA) of Universidad de Costa Rica (reference numbers 82-08 and 39-20)

## Declaration of competing interest

The authors declare the following financial interests/personal relationships which may be considered as potential competing interests:Guillermo Leon reports financial support was provided by 10.13039/100010269Wellcome Trust. All authors work at Instituto Clodomiro Picado, where the two antivenoms used in this study were manufactured.

## Data Availability

Data will be made available on request.
