# Supplementary material for: A polygeneric immunogen composed of 22 venoms from sub-Saharan African snakes to expand the neutralization scope of the EchiTAb-plus-ICP antivenom
Source: Toxicon X. 2024 Nov 16;24:100213. doi: 10.1016/j.toxcx.2024.100213 (PMC11617979; doi:10.1016/j.toxcx.2024.100213)
Supplement: Multimedia component 1 [file mmc1.docx]

Table S1: Intravenous ED_50_ and 95% CI of liquid and freeze dried antivenoms

| **Venom** | | **Standard**  **EchiTAb-plus-ICP**  Batch 6771021PALQ  Total protein 7.6 g/dL | **Expanded**  **EchiTAb-plus-ICP**  Batch 7131223PALF  Total protein 6.5 g/dL |
| --- | --- | --- | --- |
| Puff  adders | *B. arietans* | 5.0 (4.0-7.2) | 7.5 (5.9-10.7) |
|  | *B. gabonica* | 1.2 (0.7-2.3) | 1.1 (0.7-1.6) |
|  | *B. nasicornis** | < 0.5 | 0.9 (0.6-1.2) |
|  | *B. rhinoceros* | 3.0 (2.0-5.6) | 3.1 (2.5-4.5) |
| Carpet vipers | *E. leucogaster* | 2.3 (1.6-3.3) | 3.8 (2.1-7.6) |
|  | *E. ocellatus* | 5.1 (3.9-6.9) | 3.6 (2.5-4.6) |
|  | *E. pyramidum* | 2.9 (2.1-3.8) | 4.0 (3.2-5.7) |
| Mambas | *D. angusticeps** | < 0.3 | 0.5 (0.4-0.6) |
|  | *D. jamesoni** | < 0.3 | 0.7 (0.6-0.9) |
|  | *D. polylepis** | 0.1 (0.0-0.02) | 0.5 (0.3-0.5) |
|  | *D. viridis** | 0.2 (0.1-0.3) | 0.5 (0.4-0.6) |
| Spitting  cobras | *N. anchietae** | < 0.4 | 0.7 (0.5-0.9) |
|  | *N. ashei* | 0.5 (0.4-0.6) | 0.5 (0.4-0.6) |
|  | *N. katiensis** | 0.7 (0.6-1.0) | 2.0 (1.5-2.9) |
|  | *N. mossambica* | 0.8 (0.6-1.6) | 1.1 (0.8-1.6) |
|  | *N. nigricincta* | 0.7 (0.5-0.9) | 1.1 (0.8-1.3) |
|  | *N. nigricollis* | 1.0 (0.7-1.5) | 1.1 (0.8-1.4) |
| Non-spitting cobras | *N. annulifera** | < 0.4 | 0.7 (0.5-0.9) |
|  | *N. haje** | 0.1 (0.0-0.2) | 0.4 (0.3-0.6) |
|  | *N. melanoleuca** | 0.2 (0.1-0.3) | 0.8 (0.6-1.1) |
|  | *N. nivea** | < 0.3 | 0.7 (0.5-0.8) |
|  | *N. senegalensis** | 0.1 (0.0-0.2) | 0.6 (0.4-0.7) |
